# Management of cytomegalovirus reactivation and immunosuppression following uterus transplantation

**DOI:** 10.1016/j.clinsp.2026.101041

**Published:** 2026-07-08

**Authors:** Alice T.W. Song, Dani Ejzenberg, Maciana S. Silva, Vinicius R. Santos, Rafael S.N. Pinheiro, Daniel R. Waisberg, Luciana B.P. Haddad, Edson Abdala, Mario H.B. Carvalho, José M. Soares Junior, Edmund C. Baracat, Wellington Andraus

**Affiliations:** a Division of Liver and Digestive System Organ Transplants, Department of Gastroenterology, Hospital das Clínicas, Faculdade de Medicina, Universidade de São Paulo, São Paulo, SP, Brazil; b Discipline of Gynecology, Department of Obstetrics and Gynecology, Hospital das Clínicas, Faculdade de Medicina, Universidade de São Paulo, São Paulo, SP, Brazil; c Department of Infectious and Parasitic Diseases, Hospital das Clínicas, Faculdade de Medicina, Universidade de São Paulo, São Paulo, SP, Brazil; d Discipline of Obstetrics, Department of Obstetrics and Gynecology, Hospital das Clínicas, Faculdade de Medicina, Universidade de São Paulo, São Paulo, SP, Brazil

**Keywords:** Uterus transplantation, Cytomegalovirus reactivation, Infection, Immunossupression

## Abstract

Uterus transplantation (UTx) is an emerging therapeutic option for women with absolute uterine factor infertility, requiring intensive immunosuppression that increases susceptibility to opportunistic infections such as cytomegalovirus (CMV). Evidence guiding CMV management in UTx remains limited. The authors report a case of CMV reactivation in a 34-year-old woman with Mayer-Rokitansky-Küster-Hauser syndrome who underwent successful living-donor UTx. The patient received induction with methylprednisolone and basiliximab, followed by maintenance immunosuppression with tacrolimus, azathioprine, and prednisone. Antiviral prophylaxis with ganciclovir/valganciclovir was administered for three months. After prophylaxis discontinuation, routine surveillance detected CMV DNAemia approximately five months post-transplant, leading to treatment with valganciclovir and temporary postponement of embryo transfer. Following viral clearance, embryo transfer was successfully performed. During early pregnancy, low-level CMV DNAemia recurred. Given concerns about antiviral teratogenicity, management focused on the reduction of immunosuppression, including tacrolimus dose adjustment and discontinuation of azathioprine, with close monitoring via cervical biopsies. CMV DNAemia resolved spontaneously without antiviral therapy, and no rejection episodes or CMV disease occurred. This case highlights the complex balance between infection control and graft preservation in UTx recipients, particularly during pregnancy. It suggests that individualized reduction of immunosuppression may be a safe and effective strategy for managing CMV reactivation when antiviral therapy is contraindicated. Additionally, it supports the concept that the uterine graft may exhibit relatively low immunogenicity. Further studies are needed to define optimal immunosuppressive thresholds and standardized CMV management protocols in this unique transplant population.

## Highlights

• CMV reactivation after uterus transplant managed without graft rejection.
• Immunosuppression safely reduced during CMV DNAemia in early pregnancy.
• Valganciclovir is avoided during gestation due to teratogenic concerns.
• Close CMV PCR monitoring enabled timely therapeutic decisions.

## Introduction

Uterus Transplantation (UTx) represents a revolutionary therapeutic strategy for women with absolute uterine factor infertility, offering the possibility of gestation in individuals who would otherwise be unable to carry a pregnancy.^1^ Pioneering efforts, including the first successful live birth from a deceased donor UTx, have significantly advanced the field,^2^ with notable contributions also emerging from centers in Brazil, establishing initial benchmarks for patient selection and surgical outcomes.^3^^,^^4^

As a complex form of vascularized composite allotransplantation, UTx inherently requires a regimen of intensive immunosuppressive therapy. This necessity, while crucial for preventing graft rejection, also increases susceptibility to opportunistic infections, particularly Cytomegalovirus (CMV) infection or reactivation. This may lead to a spectrum of clinical manifestations, including significant fetal complications in pregnant transplant recipients.^5^^,^^6^ While CMV prevention and treatment strategies are relatively well established in other forms of solid organ transplantation, the evidence guiding CMV management specifically in the context of UTx remains sparse, given the limited number of uterine transplants worldwide. Current knowledge is largely limited to isolated case reports and small series.^7^

Here, the authors present a case of CMV reactivation following UTx in a seropositive recipient with a living donor, highlighting the diagnostic and therapeutic challenges encountered within the intricate balance of immunosuppression and the overarching goal of an intended pregnancy. Written informed consent for publication was obtained from the patient.

## Case description

A 34-year-old woman, married for 13-years and diagnosed with Mayer-Rokitansky-Küster-Hauser syndrome, presented as an otherwise healthy candidate for UTx. The living donor was her sister, who had two children and did not wish to have further pregnancies, with no history of prior abdominal surgery. Pretransplant serologies for both donor and recipient are detailed in Table 1. The patient was classified as intermediate-risk for CMV reactivation according to donor and recipient serologies.^5^

**Table 1:** Donor and recipient pretransplant serologies.

Serology	Donor	Recipient
Cytomegalovirus	IgG positive, IgM negative	IgG positive, IgM negative
Toxoplasmosis	Both IgG and IgM negative	Both IgG and IgM negative
HIV 1/2	Negative	Negative
HTLV 1/2	Negative	Negative
Herpes 1/2	IgG positive, IgM negative	IgG positive, IgM negative
Chagas	Negative	Negative
Treponema pallidum	Negative	Negative
Hepatitis A	IgG positive, IgM negative	IgG positive, IgM negative
Hepatitis B	Negative	AntiHBs positive only
Hepatitis C	Negative	Negative
Blood type	O Rh factor positive	O Rh factor positive

The transplant procedure was performed on August 17^th^, 2024. Immunosuppression protocol included 1g intraoperative methylprednisolone and a single dose of basiliximab for induction. Tacrolimus was initiated on the first postoperative day, supplemented with azathioprine 100 mg daily and prednisone 20 mg daily. Antimicrobial prophylaxis included piperacillin-tazobactam and fluconazole and was continued for 7 days post-transplant. Strongyloidiasis prophylaxis consisted of ivermectin 200 µg/kg once daily on postoperative days one and two, with a repeat dose 15-days later for another two days. Prophylactic sulfamethoxazole-trimethoprim (400/80 mg) was administered once daily for six months. Intravenous ganciclovir (5 mg/kg/day) was administered throughout the hospital stay as prophylaxis. The patient received 40 mg enoxaparin twice daily and 100 mg aspirin starting on postoperative day 1. She remained in the intensive care unit for two days, followed by five days in the transplant ward. The patient was then discharged on tacrolimus, azathioprine, prednisone, valganciclovir (900 mg once daily), and sulfamethoxazole-trimethoprim.

Protocol biopsies of the uterine cervix were performed one week after UTx, weekly during the first month, twice monthly during the second month, and monthly thereafter until gestation week-20. The initial biopsy exhibited borderline characteristics, negative C4d staining, and CD34 with endothelial reactivity. At four months post-transplant, a biopsy revealed Cervical Intraepithelial Neoplasia (CIN) Grade 2, with positive HPV DNA testing for high-risk genotypes other than 16, 18, or 45 (groups A and B). She was treated with chemical cauterization with trichloroacetic acid, with all subsequent biopsies returning to normal.

### *CMV reactivation and management*

Following the cessation of the 3-month period of valganciclovir prophylaxis, preemptive monitoring for CMV was initiated, with real-time quantitative PCR (RT-PCR, Abbott Alinithym, limit of detection 30‒100,000,000 IU/mL) performed approximately every two weeks. The patient was undergoing preparations for embryo transfer. However, on January 7^th^, 2025, approximately five months post-transplant, CMV DNAemia was detected at 1285 IU/mL (3.11 log). Consequently, valganciclovir 900 mg twice daily was initiated, and the transfer procedure was delayed. At this time, tacrolimus trough levels were 9.8 ng/mL; the dose was slightly reduced, and azathioprine was maintained.

**Fig. 1:**
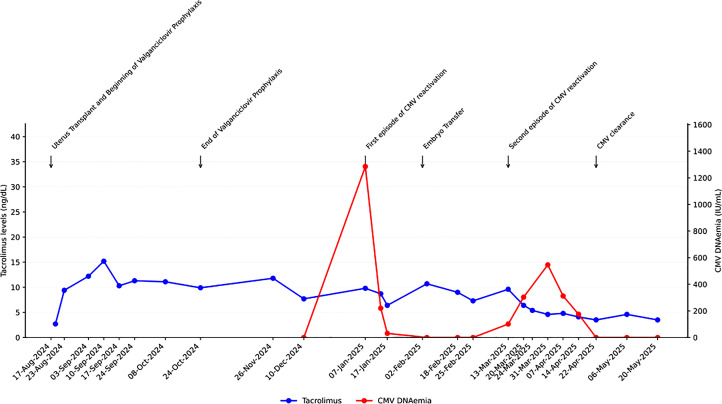
Tacrolimus (blue line, left Y-axis) serial trough concentrations (ng/mL) and RT-PCR CMV DNAemia (red line, right Y-axis) measured during the post-transplant period with arrows marking key clinical events.

CMV DNAemia and tacrolimus levels over time are depicted in Fig. 1. Given the relatively low CMV DNAemia and the need to prevent rejection episodes, tacrolimus levels were maintained at a median of 9.8 ng/mL throughout the CMV reactivation treatment period. The embryo transfer was subsequently performed on February 2^nd^, 2025, 16-days after the completion of valganciclovir treatment, and after three consecutive undetectable CMV DNAemia measurements, and preemptive monitoring was continued. Three weeks after embryo transfer, CMV DNAemia was detected at 101 IU/mL, followed by an increase to 323 IU/mL (2.51 log) one week later. In response, tacrolimus levels were tapered to a median of 4.6 ng/mL. Close monitoring for rejection with cervical biopsies continued, all of which remained normal. Azathioprine was also discontinued to further reduce immunosuppression, and prednisone was maintained at 5 mg/day. Despite the low viral load, there was significant concern because valganciclovir and ganciclovir were avoided due to concerns regarding the safety of these drugs during pregnancy. Peak CMV DNAemia of 546 IU/mL was recorded at 5-weeks post-transfer, gradually diminishing to undetectable levels by April 22^nd^, at approximately nine weeks of gestation. Beta-human chorionic gonadotropin levels of 20,894 mIU/mL on March 25^th^ confirmed the ongoing pregnancy.

## Discussion

Cytomegalovirus (CMV) reactivation represents a significant infectious complication in solid organ and vascularized composite allotransplantation, including UTx, where robust immunosuppression is required for graft survival.^8^ In the present case, the patient developed CMV reactivation and was effectively managed with a lower-than-standard immunosuppressive regimen combined with antiviral therapy. Importantly, she did not develop systemic CMV disease and showed no evidence of acute rejection throughout the clinical course.

Immunosuppression in UTx poses unique considerations not typically encountered in chronically ill SOT recipients. UTx candidates are typically young, healthy individuals, necessitating stringent monitoring, including protocol biopsies, to detect asymptomatic or subclinical rejection episodes. Episodes of graft rejection are relatively common following UTx, although most are mild to moderate in severity.^9^ Experience in uterine transplantation is limited, and therefore, there are no clear recommendations for ideal trough tacrolimus levels. The present case suggests that lower tacrolimus trough levels than those currently used may be feasible.

In the present case, due to positive CMV DNAemia occurring in the first weeks of pregnancy, trough tacrolimus levels were intentionally maintained below 5 ng/mL, yet no episodes of rejection were observed. Furthermore, this case suggests that temporary reduction of immunosuppression may be feasible in selected UTx recipients under close monitoring, since even in a young recipient, with a tacrolimus level below 5 ng/mL, and without azathioprine, the patient did not experience any rejection episodes. A major concern was the risk of transplacental CMV transmission, where the risk is estimated to be 1.4% in recurrent maternal CMV infection.^6^ Furthermore, valganciclovir is considered a potential teratogenic medication, especially during the first trimester of pregnancy, and was strictly avoided in this context. It is noteworthy, however, that an observational study did not evidence birth defects or adverse pregnancy outcomes in over 6,000 child-bearing women exposed to the drug.^10^ Some case reports have also suggested the absence of teratogenicity with oral ganciclovir in pregnant patients after kidney and liver transplantation.^11^^,^^12^ A controversial alternative, CMV-specific immunoglobulin, was not readily available in the studied institution and therefore not utilized in this patient.

The Dallas group reported a successful case of live donor UTx in a CMV D+/R- mismatch context,^7^ where the recipient presented CMV reactivation at 60,000 IU/mL after a three-month period of valganciclovir prophylaxis. She subsequently developed CMV IgG antibodies and, following embryo transfer at eight months post-UTx, did not experience further episodes of CMV reactivation. Another case of CMV reactivation during pregnancy was described in Germany, successfully managed with a one-week course of valaciclovir, despite the occurrence of massive placenta and oligohydramnios at week-19 of pregnancy.^13^

Furthermore, the CIN 2 episode may have been associated with intensified immunosuppression. However, immunosuppressive therapy was not reduced due to the need to prevent graft rejection.

The initial registry of the International Society of Uterus Transplantation indicated that most recipients were seropositive for CMV,^14^ and some groups have historically avoided transplanting grafts from CMV-seropositive into CMV-seronegative recipients.^15^ This case adds to a growing body of literature suggesting that flexibility in immunosuppression protocols may optimize outcomes in UTx recipients, especially those at high CMV risk. However, further prospective studies are needed to define the precise lower threshold of immunosuppression compatible with graft survival and reproductive success, and to establish safe protocols for managing CMV reactivation in this evolving transplant population.

### Abbreviations

UTx, Uterus Transplantation; CMV, Cytomegalovirus; RT-PCR, Real-Time Polymerase Chain Reaction; IgG, Immunoglobulin G; IgM, Immunoglobulin M; HIV, Human Immunodeficiency Virus; HTLV, Human T-lymphotropic Virus; HBs, Hepatitis-B surface antigen; DNAemia, Viral DNA detected in blood; CIN, Cervical Intraepithelial Neoplasia; ICU, Intensive Care Unit; SOT, Solid Organ Transplantation; D+/R-, Donor seropositive/Recipient seronegative; UTx Registry, International Society of Uterus Transplantation Registry; hCG, Human Chorionic Gonadotropin

### Authors’ contributions

Alice T.W. Song: Designed the structure of the report, collected clinical and laboratory data, provided direct patient care, performed literature search, wrote the first draft of the manuscript, prepared clinical descriptions, timelines, figure, and table, reviewed and approved the final manuscript.

Dani Ejzenberg: Collected surgical data, performed the surgery, provided direct patient care, integrated comments from all authors, reviewed and approved the final manuscript.

Maciana S. Silva: Collected clinical and surgical data, provided direct patient care, reviewed and approved the final manuscript.

Vinicius R. Santos: Provided direct patient care, helped interpret findings, reviewed and approved the final manuscript.

Rafael S.N. Pinheiro: Provided direct patient care, helped interpret findings, reviewed and approved the final manuscript.

Daniel R. Waisberg: Provided direct patient care, helped interpret findings, reviewed and approved the final manuscript.

Luciana B.P. Haddad: Participated in conceptualization, reviewed and approved the final manuscript.

Edson Abdala: Helped interpret findings, reviewed and approved the final manuscript.

Mario H.B. Carvalho: Provided direct patient care, helped interpret findings, reviewed and approved the final manuscript.

José M. Soares Junior: Provided direct patient care, helped interpret findings, reviewed and approved the final manuscript.

Edmund C. Baracat: Performed the surgery, provided senior oversight, edited for clarity and scientific accuracy, reviewed and approved the final manuscript.

Wellington Andraus: Conceived the case report, performed the surgery, supervised clinical decisions related to the case, provided senior oversight, collected surgical data, guided interpretation of findings, edited for clarity and scientific accuracy, reviewed and approved the final manuscript.

## Funding

The authors report no financial support for this work.

## Data availability statement

The datasets generated and/or analyzed during the current study are available from the corresponding author upon reasonable request.

## Declaration of competing interest

The authors declare that they have no competing interests relevant to this manuscript.
